# Risk of heat-related illnesses and preventive measures at mass gathering rock festivals in the summer of 2023 in Japan

**DOI:** 10.1265/ehpm.25-00350

**Published:** 2026-01-22

**Authors:** Asuka Takeda, Kaoruko Seino, Kei Shimonosono, Jun Tomio

**Affiliations:** 1 Department of Health Crisis Management, National Institute of Public Health; 2 Department of Built Environment for Health, National Institute of Public Health

**Keywords:** Extreme heat, Heat-related illness, Wet bulb globe temperature, Mass gathering, Emergency transport

## Abstract

**Background:**

Mass gatherings during the peak summer months pose a notable risk for heat-related illnesses due to extreme heat and humidity. We aimed to identify trends in preventive measures taken against heat-related illnesses at mass gathering rock festivals in Japan during the summer of 2023, in terms of associations with online sources regarding the event details and heat risk communication; daily maximum wet bulb globe temperature (WBGT) values; and emergency heat-related illness transport data.

**Methods:**

Four rock festivals (festivals A–D), held in July and August 2023, were analyzed. Publicly available online sources were reviewed to examine the event details, heat risk communication, and preventive measures. Daily maximum WBGT values were obtained from the meteorological observation points closest to each festival venue. Emergency heat-related illness transport data were assessed to identify trends during the rock festivals.

**Results:**

Three festivals (festivals A, B, and D) recorded daily maximum WBGT values at or above the “Danger” threshold (≥31 °C), while festival C reached the “Severe Warning” level (≥28 °C and <31 °C). Despite the high WBGT, no heat advisories were issued on the websites of festivals A and B. Festival C gave a basic advisory on heat precautions. Festival D implemented more comprehensive countermeasures, including public warnings and recommendations to carry hats, towels, and portable fans. Notably, emergency transport for heat-related illnesses increased in the regions hosting festivals B and D.

**Conclusions:**

All festivals posed substantial heat-related health risks. Enhanced public risk communication and consistent preventive measures are essential for mitigating preventable incidents during mass gatherings in extreme heat.

## Introduction

Rising temperatures and frequent extreme heat events have become pressing public health challenges, exacerbated by the ongoing effects of climate change. These conditions pose heightened risks at mass gathering events [1]. The World Health Organization defines mass gatherings as events characterized by a concentration of people at a specific location for a defined purpose that may strain local resources and health systems [2]. Summer rock festivals are particularly prone to creating hazardous environments owing to crowd density, prolonged exposure to solar radiation, and limited access to cooling and hydration resources [3]. While heat-related illnesses are well documented in sporting events, little is known regarding the risks and mitigation efforts associated with rock festivals [4]. Moreover, Japan hosts approximately 50 rock festivals from July to August in recent years, with audience sizes varying from several hundreds to tens of thousands. While outdoor rock festivals are also prevalent in Western countries during the summer and autumn months, Japan’s hot and humid climate present unique environmental challenges in managing heat-related risks. Recent news reports have documented instances of heat-related illness at mass gathering rock festivals in Japan, underscoring the need for research on environmental risks and preventive measures at such events. In Japan, summer typically spans from June to September, with July and August being the hottest months. In recent years, daily maximum temperatures have frequently exceeded 35 °C, even surpassing 40 °C in some areas. During this period, high temperatures combined with elevated humidity notably increase the risk of heat-related illnesses.

This study aimed to describe the environmental risk of heat-related illnesses and assess the preventive measures undertaken at major rock festivals held in Japan during the summer of 2023.

## Methods

Four of the most attended rock festivals held in Japan during July and August 2023 were selected for analysis, hereafter referred to as festivals A through D. These four festivals were chosen because they are among the largest and most widely recognized in Japan, and they attract tens of thousands of attendees, representing typical mass gathering rock festivals. Information detailing each festival was extracted from official websites and publicly available sources accessed on October 11, 2023. Data for event dates, venue locations (prefectures), indoor and outdoor settings, and total attendance were collected. Additional items reviewed included the presence or absence of general advisories related to heat-related illnesses, reminder of heat mitigative measures (e.g., hydration reminders or shaded rest areas), policies on personal beverage carry-in, permission for parasol use, and the number of designated first-aid stations available at the venue.

To assess the environmental risk of heat-related illness, daily maximum wet bulb globe temperature (WBGT) values were obtained from the meteorological observation points closest to each festival venue, based on data published by the Ministry of the Environment, Japan (MOE). The WBGT reflects the combined effects of humidity, solar radiation, and ambient air temperature. In Japan, the MOE uses the daily maximum WBGT as the key threshold value in the Heat Stroke Alert and Special Heat Stroke Alert systems. According to the heat illness prevention guideline for sports activities developed by the Japan Sports Association and the guideline for daily life issued by the Japanese Society of Biometeorology [5, 6], WBGT values between 28 °C and less than 31 °C are classified as “Severe Warning”, and values of 31 °C or above are designated as “Danger”, indicating a high risk of heat-related illness based on expected levels of physical activity in sports and daily life.

As the number of heat-related illness cases among attendees at each festival was not available, the number of emergency medical transports due to suspected heat-related illnesses was examined using daily surveillance data published by the Fire and Disaster Management Agency of Japan to evaluate potential health impacts at the population level [7]. This study conducted is an ecological analysis and does not conclusively determine whether patients who received emergency transport for treating heat-related illness actually attended the rock festivals. Trends in emergency transport were analyzed for the one-month period surrounding each festival, with particular attention paid to any increases coinciding with the event dates.

Ethical approval was not required for this study, as it did not involve human subjects or identifiable personal information.

## Results

Festival A and B websites lacked heat illness advisories, while festival C website included a simple general advisory to take precautions against heat-related illnesses. In contrast, festival D provided warnings, hydration reminders, and information regarding rest areas and first-aid facilities; furthermore, attendees were encouraged to carry heat-mitigation items such as hats, towels, and portable fans. However, specific data on patient numbers and diagnoses at onsite first-aid stations were not disclosed.

Based on the thresholds for heat-related illness risks defined by daily maximum WBGT levels, readings exceeded 31 °C—the “Danger” level—during festivals A, B, and D (Table 1). In contrast, festival C that took place in Hokkaido, situated in the northernmost part of Japan, and characterized by lower temperatures and humidity compared to other regions, remained within the “Severe Warning” range (≥28 °C and <31 °C).

**Table 1:** Overview of heat-related illness risks and mitigation measures at each rock festival in the summer of 2023 in Japan.

**Festival name**	**Date**	**Event hours**	**Prefecture** **(latitude, longitude)**	**Total attendance** **(approximate)**	**General advisories against HRI**	**Descriptions of ** **specific preventive measures against ** **HRI**	**Carry-in of beverages**	**Use of ** **parasols**	**No. of ** **first-aid stations**	**Daily maximum WBGT** **(°C)**	**Time of daily maximum WBGT**
A	28 July (Fri)	8:30–29:00	Niigata (36.7918, 138.7846)	29,000	Absent	Absent	Glass bottles and cans are prohibited	Prohibited	4	31.1	14:00
	29 July (Sat)	8:30–29:00		38,000						30.3	13:00
	30 July (Sun)	8:30–29:00		29,000						29.6	16:00

B	05 August (Sat)	11:50–20:40	Chiba (35.5747, 140.1242)	53,000	Absent	Absent	Glass bottles and cans are prohibited	Permitted outside the stage area	5	32.2	12:00
	06 August (Sun)	11:50–20:40		53,000						30.6	12:00
	11 August (Fri)	11:50–20:40		53,000						30.0	10:00
	12 August (Sat)	11:50–20:40		53,000						30.7	15:00
	13 August (Sun)	11:50–20:40		53,000						31.0	8:00

C	11 August (Fri)	13:00–25:00	Hokkaido (43.1760, 141.3016)	32,000	Present	Absent	Glass bottles and cans are prohibited	Permitted outside the stage area	1	30.2	12:00
	12 August (Sat)	12:00–29:00		32,000						29.6	14:00

D (Tokyo)	19 August (Sat)	9:15–27:50	Chiba (35.6452, 140.0309)	62,500	Present	Present	Glass bottles and cans are prohibited	Permitted outside the stage area	5	32.1	12:00
	20 August (Sun)	9:30–21:40		62,500						31.7	12:00

D (Osaka)	19 August (Sat)	10:10–21:40	Osaka (34.8067, 135.5301)	45,000	Present	Present	Glass bottles and cans are prohibited	Permitted outside the stage area	5	31.3	14:00
	20 August (Sun)	10:10–21:55		45,000						32.5	13:00, 14:00

Note: 1) HRI: heat related illnesses, WBGT: wet bulb globe temperature

2) August 11 (Fri) is a national holiday in Japan.

**Fig. 1:**
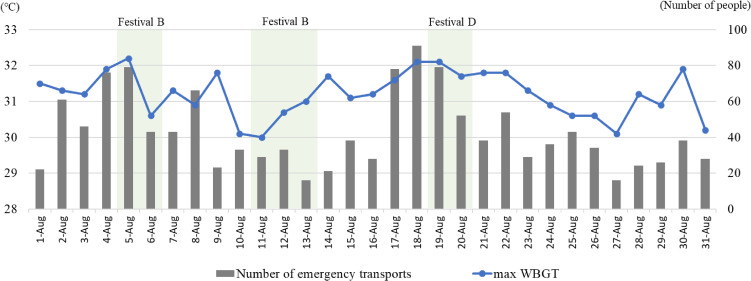
Trends in heat-related emergency transports and daily maximum WBGT, Chiba Prefecture, August 2023. The left vertical axis indicates the daily maximum wet bulb globe temperature (WBGT), while the right vertical axis represents the number of emergency transports for suspected heat-related illnesses.

On August 5 and 19 (opening days of festivals B and D), Chiba Prefecture recorded an increased number of emergency heat-related illness cases, suggesting a potential association (Fig. 1). Notably, in August 2023, Chiba Prefecture experienced three days wherein the daily maximum WBGT exceeded 32 °C with more than 70 people transported daily by ambulance for suspected heat-related illnesses.

## Discussion

The findings of this study highlight the substantial environmental risks of heat-related illnesses at all four rock festivals, particularly during peak daylight hours. In addition to high ambient temperatures, rock festivals present unique circumstances that can intensify heat stress, such as densely-crowded standing areas that limit movement, prolonged outdoor waiting to secure good viewing positions, and alcohol consumption that may worsen dehydration and hinder early recognition of symptoms of heat-related illness. Moreover, the variation in preparedness, particularly in public communication, suggests a lack of standardization in risk-mitigation practices.

With the increasing frequency and intensity of extreme heat events in Japan, mass gathering event organizers must adopt consistent and evidence-based heat risk management [8]. Although audience demographics may vary depending on the genre, rock festivals generally attract younger crowds. These attendees may be less inclined to purchase drinks onsite, particularly when prices are higher than usual, which could contribute to inadequate hydration. They may also consume alcoholic beverages while dancing [9], leading to an elevated or euphoric state that can impair their recognition of early symptoms of heat-related illness. Shade provision, hydration infrastructure, and adequate medical facilities are essential components of preparedness [10]. As there are limits to the measures that event organizers can implement, effective messaging to promote individual-level heat illness prevention is essential. In addition to individual- and organizer-level measures, adjusting the timing and duration of outdoor events and performances to avoid peak heat hours (typically from noon to mid-afternoon) should be considered in future event planning. Establishing clear criteria for postponement or cancellation of events and rock festivals during extreme heat warnings can help reduce health risks. Furthermore, the systematic collection and publication of health incident data from such events would enhance future risk assessment and response planning.

Encouragingly, in 2024, some rock festivals investigated in this study had expanded shaded rest areas and designated more spaces where parasols were permitted. These changes reflect a growing recognition of the importance of respecting and supporting individual-level heat mitigation strategies within the event environment.

Summer rock festivals offer great enjoyment for music enthusiasts, but they also pose a considerable risk of heat-related illnesses. Proactive communication and consistent application of heat mitigation measures are urgently required to safeguard the public during mass gatherings under extreme heat conditions.

### Limitations

This study has a few limitations. First, data regarding the number and clinical characteristics of patients who visited on-site medical facilities at each rock festival were not available. Second, in the case of Chiba Prefecture where the number of emergency transports due to suspected heat-related illnesses was particularly high during the festival period, the available data covered the entire prefecture rather than being limited to the immediate vicinity of the event venue. Additionally, the survey period coincides with a period of increased outdoor leisure and shopping activities. The potential impact of these concurrent outdoor activities on the risk of heat-related illness was not considered in this study, which could have influenced the number of emergency transports observed during the festival period. Third, daily maximum WBGT values were not directly measured at the festival venues on the respective event days; instead, data were taken from the nearest meteorological observation sites.

## Conclusion

Mass gathering rock festivals pose substantial heat-related health risks. Enhanced public risk communication and consistent preventive measures are essential for mitigating preventable incidents during mass gatherings in extreme heat.

## Abbreviations

WBGT: wet bulb globe temperature

## Declarations

### Availability of data and materials

The datasets used and analyzed during the current study are available from the corresponding author on reasonable request.
